# Development of primary human pancreatic cancer organoids, matched stromal and immune cells and 3D tumor microenvironment models

**DOI:** 10.1186/s12885-018-4238-4

**Published:** 2018-03-27

**Authors:** Susan Tsai, Laura McOlash, Katie Palen, Bryon Johnson, Christine Duris, Qiuhui Yang, Michael B. Dwinell, Bryan Hunt, Douglas B. Evans, Jill Gershan, Michael A. James

**Affiliations:** 1 Department of Surgery, Medical College of Wisconsin, Milwaukee, WI 53226 USA; 2 Department of Pediatrics, Medical College of Wisconsin, Milwaukee, WI 53226 USA; 3 Department of Pathology, Medical College of Wisconsin, Milwaukee, WI 53226 USA; 4 Department of Microbiology and Immunology, Medical College of Wisconsin, Milwaukee, WI 53226 USA; 5 Surgical Oncology, 4850 TBRC, Medical College of Wisconsin, 8701 Watertown Plank Road, Milwaukee, WI 53226 USA

**Keywords:** Pancreatic Cancer, PDAC, Organoid, Organotypic culture, Microenvironment, Tumor stroma, Tumor immunology, TILs, CAFs

## Abstract

**Background:**

Patient-derived tumor models are the new standard for pre-clinical drug testing and biomarker discovery. However, the emerging technology of primary pancreatic cancer organoids has not yet been broadly implemented in research, and complex organotypic models using organoids in co-culture with stromal and immune cellular components of the tumor have yet to be established. In this study, our objective was to develop and characterize pancreatic cancer organoids and multi-cell type organotypic co-culture models to demonstrate their applicability to the study of pancreatic cancer.

**Methods:**

We employed organoid culture methods and flow cytometric, cytologic, immunofluorescent and immunohistochemical methods to develop and characterize patient-derived pancreatic cancer organoids and multi-cell type organotypic co-culture models of the tumor microenvironment.

**Results:**

We describe the culture and characterization of human pancreatic cancer organoids from resection, ascites and rapid autopsy sources and the derivation of adherent tumor cell monocultures and tumor-associated fibroblasts from these sources. Primary human organoids displayed tumor-like cellular morphology, tissue architecture and polarity in contrast to cell line spheroids, which formed homogenous, non-lumen forming spheres. Importantly, we demonstrate the construction of complex organotypic models of tumor, stromal and immune components of the tumor microenvironment. Activation of myofibroblast-like cancer associated fibroblasts and tumor-dependent lymphocyte infiltration were observed in these models.

**Conclusions:**

These studies provide the first report of novel and disease-relevant 3D in-vitro models representing pancreatic tumor, stromal and immune components using primary organoid co-cultures representative of the tumor-microenvironment. These models promise to facilitate the study of tumor-stroma and tumor-immune interaction and may be valuable for the assessment of immunotherapeutics such as checkpoint inhibitors in the context of T-cell infiltration.

## Background

Conventional two-dimensional culture of tumor cells has been a staple of in-vitro testing for both basic science and clinical applications. However, these cultures have proven not to be representative of tumor heterogeneity, structure, cell to cell contact, or gene expression. For these reasons, they provide incomplete models of normal and pathologic processes in the context of human tissues.

Organotypic cultures are those that recapitulate the complex cellular environment of the tissue of origin, including parenchymal and stromal components [1]. These systems include cell-cell, cell-extracellular matrix (ECM), and ideally, cell-immune interactions. In the context of cancer, ECM remodeling, pro-tumorigenic stromal cell factors, and anti-tumor immune infiltration are critical players in the growth and spread of human cancer.

Ex-vivo culture of organs or organ slices enable limited experimentation in the context of stromal tissues, although viability is typically short-lived and these cultures cannot be expanded, banked, or genetically manipulated. These models are not amenable to experimentation on immune, tumor or stromal cell migration. Additionally, viability of individual cell types within a tissue may be disparate in these cultures.

Tissue organoid cultures offer the advantages of expandability from stem cells derived from organs and primary tissues, genetic manipulability [2], long term storability, and the ability to xenograft into animal models. Importantly, these cultures retain physiologic tumor/organ architecture and cell-cell and cell-stroma interactions.

Three-dimensional organotypic models of the tumor environment are offering a new paradigm both for guidance of personalized therapy and for preclinical evaluation of novel therapeutics. While implementation of these precision 3D in-vitro models has gained some traction in certain diseases, preclinical modeling in pancreatic cancer has thus far been limited to patient-derived mouse xenografts (PDX). The utility of PDX models has been limited to preclinical evaluation of therapeutics [3] and the success of their engraftment has been shown to correlate with the extent of pre-harvest therapy. Heavily pretreated tumors are often not successful as xenografts [4]. Further, these models are too time-consuming to be highly valuable for guidance of personalized therapy during the short duration of disease progression for patients with pancreatic cancer. Organoids are cultured directly from patient samples within days, rather than passaging through mice, which can take months. Primary cultures of this nature are much more representative of tumor tissues than those expanded in mice in terms of heterogeneity, polarity, cell-cell contact and structure when grown in 3D. Therefore, it is of great value to develop representative in-vitro organotypic models of pancreatic tumors and their microenvironment that can be rapidly constituted and propagated as a resource for basic and translational science.

Pancreatic ductal adenocarcinoma is particularly influenced by a dense stroma containing cancer-associated fibroblasts (CAFs), which can contribute tumor-promoting growth factors and cytokines [5], and immune infiltrates [6]. Stromal cells constitute a significant portion of such tumors, and inflammation is closely linked to pancreatic cancer pathogenesis. In particular, pancreatic stellate cells are prevalent in the tumor microenvironment, which are myofibroblast-like and function normally in tissue repair [7]. Inflammatory fibroblasts may also be a distinct population of stromal cells that contribute to tumor growth [8]. We have recently reported chemokine recruitment of stellate cells to the pancreatic cancer microenvironment [9]. Inflammation (pancreatitis) is closely linked to pancreatic tumorigenesis [10, 11]. Immune infiltrates are prevalent in pancreatic tumors, but the environment is highly immunosuppressive [12]. The recruitment of regulatory T cells into the tumor can be influenced by CAFs [13]. In the context of in vitro models, we have previously demonstrated the use of collagen matrices to study the migration of lymphocytes [14].

Patient-derived organoid cultures have recently been described as a potentially powerful research tool in the field of pancreatic cancer [2, 15–17]. Further, the co-culture of mouse model-derived organoids and fibroblasts was also recently described [8]. Patient-derived organoids may offer a more representative model of disease as well, providing a genetically manipulable system that may be propagated, stored, and transferred into complex 3D in-vitro or murine in-vivo models. They are also representative of disease stage, tumor tissue architecture, cytology and pathology [15]. However, the utility of human pancreatic cancer organoid cultures directly derived from surgical specimens for practical research applications has not been further described beyond these initial studies to our knowledge.

Herein, we report the establishment, characterization and banking of human tumor-derived organoid cultures directly from resected primary and metastatic tumors, ascites, and rapid autopsy specimens, as well as patient-matched cancer-associated fibroblasts and peripheral blood lymphocytes. We demonstrate that spheroids derived from cell lines established in monolayer or passaged as mouse xenografts are phenotypically distinct from primary organoids. We demonstrate disease-representative modeling of the pancreatic cancer microenvironment using three-dimensional organotypic co-culture of primary organoid, stromal and immune components, and lymphocyte infiltration into extracellular protein matrices that is dependent upon tumor organoids in these models. These organotypic cultures were established within days of collection and offer a powerful method for precision science and translational medicine.

## Methods

### Surgical specimens and regulatory compliance

Surgical specimens, immediate post-mortem specimens, or ascites were delivered from the operating room/anatomy lab on ice as rapidly as possible (always in less than 2 h of devascularization). Solid tissues were transported in primary cell culture media. The Pancreatic Cancer Program infrastructure provided support for routine patient consent to acquire blood and tumor specimens. At the time of consent, the purpose of the Pancreatic Cancer Biorepository was explained in detail. Upon enrollment, tissue which was not needed for clinical care was made available to the Biorepository at the time of surgery or tumor biopsy, including adjacent normal pancreas. Peripheral blood was processed into peripheral blood mononuclear cells (PBMCs), serum, and plasma within 2 h. PBMCs were obtained by Ficoll® density gradient centrifugation and frozen in cyrovials at a concentration of 3 × 10^6^ cells/mL in liquid nitrogen. As part of the rapid autopsy program, patients with terminal pancreatic cancer made a decision to donate their organs for tumor banking post-mortem. The autopsy was coordinated to occur as soon as possible (usually within 1-2 h) after death and primary and metastatic tumors were retrieved and banked.

### Patient-derived and established PDAC cell lines

MCW670 cells were established at the Pancreatic Cancer Biorepository at MCW and maintained in DMEM/F12 with 6% FBS and supplements. Cell lines were generated from heterotopic murine xenografts established from primary and metastatic human pancreatic cancer specimens. Cell lines were established after enzymatic digestion of the xenografts. Mouse CD326- MHC Class I+(H-2Kd) cells were eliminated from the cell lines by flow cytometric FACS sorting using human-specific CD326 (EpCAM) and murine-specific MHC Class I (H-2Kd) antibodies (eBioscience, San Diego, CA). Short tandem repeat (STR) profiling was performed using 17 STR loci plus the gender determining locus using the commercially available PowerPlex 18 D Genetic Analyzer. Data were analyzed using GeneMapper IDX v1.2 (Applied Biosystems). Samples did not match any cell line in either the American Type Culture Collection database. Cell lines were characterized by immunohistochemistry (IHC) for gastroenteropancreatic and hepatobiliary epithelial (CK19) and pancreatic (PDX-1) markers, doubling time, colony forming efficiency, and in-vivo tumorigenicity. Mutations in KRAS and TP53 were assessed using Sanger sequencing. The cell line harbors KRAS G12A mutations. Panc1 and A549 cells were cultured in RPMI1640 plus 10% FBS (Life Technologies, Carlsbad, CA) and were authenticated within 6 months of experiments.

### Media

Primary cell culture medium: (Dulbecco’s Modified Eagle’s Medium Nutrient Mixture F-12 (Ham), (ThermoFisher). Per 500 ml medium: 30 mL FBS (ThermoFisher), 5.5 ml Penicillin/Streptomycin (ThermoFisher), 500 μl EGF Human recombinant (100μg/ 1 ml dH20) (ThermoFisher), 2 ml Bovine Pituitary Extract (ThermoFisher), 20 ml Hydrocortisone (1 mg dissolved in 1 ml ETOH plus 19 ml dH2O) (Sigma), 70ul Insulin Human Recombinant (ThermoFisher), Glutamax I (ThermoFisher). Organoid Wash Medium: Advanced DMEM/F12, HEPES (1 M), GLUTAMAX, 2.5% Heat Inactivated FBS (ThermoFisher), 1X Primocin (Invivogen). Basic Organoid Medium: Intesticult Stem Cell Media (Stem Cell Technologies) with included supplements, Primocin. Organoid Growth Medium (OGM): 25 mL Basic Organoid Media, 25 μL A83-01 (Sigma) 0.2 mg/mL(0.5 mM) in DMSO, 250 μL (2.5 μg) hEGF (ThermoFisher) 5 μg vial reconstituted in 500 μL Basic Organoid Media (remaining 250 μL frozen for future use). 250 μL (2.5 μg) hFGF-10 (ThermoFisher) 5 μg vial reconstituted in 500 μL Basic Organoid Medium (remaining 250 μL frozen for future use), 25 μL Gastrin I (Sigma) 0.021 mg/mL(10uM) in 0.1% NaOH, 75 μL N-acetylcysteine (Sigma) 81.5 mg/mL(500 mM) in dH_2_0, 250 μL Nicotinamide (Sigma) 122 mg/mL(1 M) in dH_2_0, 500 μL B27 supplement (ThermoFisher). For initial seeding, splitting or thawing, 1:1000 Y-27632 (Rho Kinase Inhibitor) (Sigma) 3.6 mg/mL (10.5 mM) in dH20 was added to OGM. OGM was filtered through a 0.22 μM Steriflip filter (Millipore). For normal epithelial tissue organoids, 1:1000 PGE2 (Sigma) 0.352 mg/mL (1 mM) in dH_2_0 was added to OGM. Digestion Medium: 10 mg Collagenase II (ThermoFisher) and 40 mg Dispase II (ThermoFisher) was added to 8 mL of Organoid Wash Media per specimen to be digested.

### Organoid culture from primary tumor tissues and co-culture

Surgical specimens were minced into 1 mm^3^ fragments in a sterile tissue culture dish with sterile forceps and #10 scalpel. Minced specimens were transferred to 8 mL of 37 °C Digestion Media in a 15 mL conical tube and placed on a rocker at 37 °C for 2 h. Tubes were allowed to settle upright at room temperature for 2 min. Supernatants were transferred to a new 15 mL conical tube containing 6 mL cold Wash Media, leaving any solids behind. Tubes were centrifuged at 150 x g, 4 °C, 5 min. Supernatants were aspirated and pellets washed twice with 6 mL Wash Media and centrifugation as above. Pellets were resuspended in 1.5 mL 37 °C TryplE (ThermoFisher). If visible clumping was observed, 10 μL of DNAse I (Sigma) 5 mg/mL in sterile DPBS was added. Tubes were placed on a rocker at 37 °C for 15 min. Tubes were centrifuged as above and washed once in Wash Media as above. Supernatants were thoroughly aspirated without disturbing the pellet, leaving up to 20 μL of Wash Media. Pellets were resuspended in 100 μL liquefied Matrigel (Corning) at 4 °C. Aliquots of 35 μL of resuspended cells in Matrigel were plated in triplicate as droplets in the centers of wells of a 24-well tissue culture plate, warmed to 37 °C and kept on a 37 °C warming block. Plates were incubated at 37 °C, 5% CO_2_ for 15 min to solidify Matrigel droplets. Droplets were overlaid with 500 μL of 37 °C OGM with Rho kinase inhibitor and incubated at 37 °C, 5% CO2 in a humidified incubator. For established and primary cell lines (Panc1, MCW670, A549) organoid suspensions were overlaid with either OGM or RPMI1640 with 10% FBS. Cultures were media changed every 2-3 days by careful aspiration of the media overlay and gentle administration of fresh media to the side of the well. For ascites, up to 150 mL was centrifuged at 150 x g, 4 °C, 5 min. Pellets were resuspended in 5 mL ACK lysing buffer (ThermoFisher) and incubated at 4 °C, 5 min to lyse red blood cells. No digestion was performed. Pellets were washed in 6 mL Wash Media twice, removing mucinous or solid components with a 1000 μL pipette and tip. Cell pellets were suspended in Matrigel and plated in 35 μL droplets as described above. The volume of Matrigel that pellets were resuspended in depended on the size of the pellets, with the pellet volume not exceeding 25% of the total resuspended volume. For fibroblast co-culture, 5 × 10^5^ patient-matched CAFs per well were suspended in Matrigel and plated with organoids. For lymphocyte co-culture, 500,000 CD3+ T lymphocytes per well were suspended in 500 μL OGM and added to organoids in Matrigel domes or empty Matrigel domes. After 72 h in culture, lymphocytes in media were removed for flow analysis and immunofluorescence was conducted on remaining organoids and lymphocytes in Matrigel domes as described below.

### Organoid splitting and freezing

Cultures were split when organoids became large (~ 300 uM) or over-confluent (once every few days to weeks depending on growth rate). Media was aspirated away from Matrigel domes. Matrigel was broken up and dissolved in 500 μL cold (4 °C) PBS with a 1000 μL pipette and tip and added to a 15 mL conical tube with 8 mL cold PBS. Tubes were centrifuged at 300 x g, 4 °C, 5 min. All but 2 mL supernatant was aspirated. Visible organoids were broken up by gentle passage through a 23 g 1″ needle on a 3 mL syringe. Tubes were centrifuged at 300 x g, 4 °C, 5 min. Supernatants were thoroughly aspirated, leaving the pellet and up to 20 μL of supernatant. If cells were to be frozen, pellets were resuspended in 1 mL Restore freezing media (ThermoFisher) and frozen in 0.5 mL cryovial aliquots at − 80 °C for at least 4 h before transferring to liquid nitrogen. If cells were to be re-plated, pellets were resuspended in Matrigel and plated as above. Wells were overlaid with 500 μL of 37 °C OGM with Rho kinase inhibitor and incubated at 37 °C, 5% CO2 in a humidified incubator.

### Isolation and characterization of lymphocytes from blood

Human T-cells were isolated from human frozen blood buffy coats using a MagniSort Human T Cell Enrichment Kit (ThermoFisher) and quantified pre- and post-enrichment by flow cytometry gated on CD3 positivity. CD4+ and CD8+ populations were measured by flow cytometry. T-cells were seeded into 24-well plates at 1 × 10^6^ cells per well in either RPMI or OGM in a humidified CO2 incubator at 37 °C. For activation, Dynabeads Human T activator CD3/CD28 (ThermoFisher) were added at a 1:1 bead:cell ratio per manufacturer’s instructions.

### T cell cultures and flow cytometry

Human T-cells were purified from frozen buffy coats and isolated by negative selection using the MagniSort Human T Cell Enrichment Kit (Thermofisher). T cells were seeded into wells of a 48 well plate at a density of 1 × 10^6^ cells/well and activated at a 1:1 ratio with Human T activator CD3/CD28 conjugated Dynabeads (ThermoFisher). Cells were cultured in a humidified CO2 incubator at 37 °C for 6 days in either organoid media or RPMI plus 10% fetal bovine serum (FBS), 100 U/ml penicillin, 100 μg/ml streptomycin, 1 mM sodium pyruvate, 10 mM Hepes buffer, 69 mM L-arginine and 50 μg beta-mercaptoethanol. After 6 days in culture, cells were stained with fluorochrome-conjugated monoclonal antibodies: anti-CD3 (SK7), anti-CD4 (OKT-4) and anti-CD62L (DREG-56) were obtained from eBioscience; and anti-CD8 (RPA-T8) was obtained from BD Biosciences. Cells were stained with 7-aminoactinomycin (7AAD, eBioscience) as a viability marker. Flow cytometry was performed on a LSRII flow cytometer. (BD Biosciences), and the data were analyzed using FlowJo software version 10 (FlowJo, LLC).

### Isolation of CAFs

Following trypsin digestion of the tumor specimen described above, the tube was allowed to sediment for 2 min and the pellet was plated on 10 cm tissue culture dish. Spindle-shaped, fibroblast-like monolayer outgrowth was passaged by trypsinization for 2 min at 37 °C and gentle rinsing. After 3 passages, immunofluorescence for vimentin and αSMA confirmed fibroblast and activated fibroblast phenotypes, respectively. Sanger sequencing of the KRAS gene showing wild-type G12 confirmed no epithelial contamination.

### Immunofluorescence

Media was aspirated away from Matrigel domes. Matrigel domes were washed with 500 μL room temperature PBS. Wells were fixed with 4% paraformaldehyde in PBS for 30 mins at room temperature. Wells were rinsed 3 × 10 min with 500 μL 100 mM glycine in Tris pH 7.4. Cells were permeabilized with 500 μL 0.5% Triton X-100 in PBS at room temperature for 5 min. Wells were washed in IF wash buffer (0.1% BSA, 0.2% Triton X-100, 0.05% Tween 20) 3 × 10 min. Wells were incubated with 500 μL of IF wash buffer with 1% BSA to block for 1 h at room temperature. Block buffer was aspirated and wells were incubated with primary antibody (mouse anti-αSMA, AbCam; mouse anti-Maspin, BD-Pharmingen; Rabbit anti-PDX1, Cell Signaling Technology; mouse anti-CK19, AbCam) 1:200 in 500 μL block buffer for 1 h at room temperature. Wells were washed 3 × 20 min in IF wash buffer. Wells were incubated with secondary antibody (Anti-Mouse Alexafluor Texas Red, ThermoFisher or Anti-Rabbit Alexaflour 488) 1:500 in 500 μL block buffer for 1 h at room temperature. Wells were washed 3 × 20 min. Wash was removed thoroughly and 150 μL of SlowFade anti-fade mountant with Dapi (ThermoFisher). Organoids were observed and micrographed on a Zeiss inverted microscope with fluorescence and Zen analysis software (Zeiss).

### Organoid histology

Organoids grown in Matrigel on coverslips submitted for histology and immunohistochemistry were initially fixed in 4% PFA for 1.5 h. After fixation, the organoids were PBS washed and hematoxylin inked. The organoids were pre-embedded in Histogel (Richard Allen HG-4000-012) and returned to fixative in 4% PFA overnight. Organoids embedded in Histogel were processed with an automated tissue processor (Sakura VIP6) and embedded into a paraffin block (Sakura Tissue-Tek TEC10). Samples were sectioned at 4 μm (Microm HMS355S) onto poly-l-lysine coated slides and air-dried 45 °C overnight for any subsequent immunohistochemistry or routine H&E staining.

### Immunohistochemistry

An optimal immunostaining protocol was developed with the use of a Leica-Bond Max Immunostaining platform. All slides were dewaxed prior to their optimal antigen retrieval protocol. PDX-1 (Cell Signaling #5679 1:50), Maspin (BD Pharmigen, 554292 1:100) and CLPTM1L (Sigma HPA014791, 1:400) were dewaxed prior to their optimal epitope retrieval protocol. Maspin and CLPTM1L antibodies required high pH epitope retrieval (Epitope Retrieval Solution 2, AR 9640). PDX1 performed optimally with citrate buffer ph 6 (Epitope Retrieval 1 AR9961). Muc5ac (ThermoFisher MS-145-PO, 1:200) did not require epitope retrieval. All antibodies were detected and visualized using Bond Polymer Refine Detection System (DS9800) with the addition of a DAB Enhancer (AR9432), using the Modified F protocol (primary antibody incubation 30 min-1 h). All slides were counter-stained with hematoxylin and coverslipped using a synthetic mounting media. Omission of the primary antibody served as negative control.

### Immunofluorescence on paraffinized slides

All slides for fluorescence were deparaffinized and antigen retrieved in pH 6 citrate buffer for a total of 40 min. Staining was performed on a DAKO Autostainer Plus immunostaining platform. After protein blocking (30 min, DAKO X0909) the anti-Laminin-alpha5 1:100 Abcam 77175 was incubated overnight at 4 °C. Goat anti-Mouse Alexa Fluor-488 at a 1:500 for 45 min was used to visualize Laminin-alpha5. Nuclei were stained with DAPI (Sigma, D8417). Negative control staining was done with isotype control primary antibodies. Imaging was performed on a Nikon fluorescent microscope with Elements software.

### PCR

DNA was extracted from organoid pellets by Qiagen DNeasy Kit. Amplification was performed on a MyIQ cycler (BioRad): 95 °C for 5 min; 40 cycles of 95 °C for 30 s, 55 °C for 30 s, 72 °C for 30 s; 72 °C for 5 min; 10 °C hold. Ion Torrent primers for K-Ras, exon 2: For- CCATCTCATCCCTGCGTGTCTCCGACTCA, Rev- CCTCTCTATGGGCAGTCGGTGATTATCGTCAAGGCACTCTTGC. Sanger sequencing was performed by Functional Biosciences, Inc.

## Results

### Establishment and expansion of human PDAC organoids, cell lines, and CAFs

**Fig. 1:**
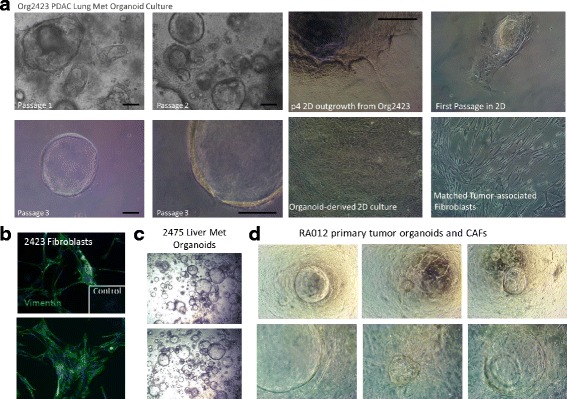
Organoid and CAF Culture from Primary and Metastatic Human Pancreatic Tumors. **a** Micrographs of a human PDAC lung metastasis thro ugh 3 passages in organoid culture, monolayer outgrowth of tumor and fibroblast cultures. **b** Immunofluorescent staining of Vimentin in patient matched fibroblasts. **c** Micrographs of organoid culture of a human PDAC liver metastasis. **d** Micrographs of organoid culture of a primary human PDAC tumor collected from a rapid autopsy with associated fibroblasts. Bars, 100 μM

To establish a bank of patient-derived organotypic tumor cultures, we have cultured organoids from the tumor tissues of 28 patients with pancreatic cancer, including primary and metastatic tumors, ascites and rapid autopsy specimens (Table 1). As an indication of the variation in proliferative rates of organoid cultures between tumor specimens, the time to the first splitting of organoids (when organoids grew to 300 μM or became densely packed) ranged from 3 days to 2 weeks. Figure 1 depicts organoid cultures from multiple patients including tissue bank numbers 2423 from a lung metastasis, 1710 from ascites, and RA012 derived from a primary tumor collected from a rapid autopsy. Organoids derived from surgically-obtained pancreatic cancer specimens (primary and metastatic pancreatic tumors) took an average of 7 days to proliferate to the point that required splitting of cultures. Samples isolated from omentum and peritoneal metastases took 3 and 5 days, respectively (Table 2). Rapid autopsy specimens took longer to grow (20-27 days to splitting), although those specimens were stored at 4 °C overnight (when the autopsy was performed in the evening/night) and may have lost viability during that time or during transport for autopsy. Also notable, was the slower growth of KRAS wild-type organoids, with an average time to splitting of 21 days compared to 4 days for KRAS (G12) mutant organoids (Table 2). Organoid cultures were typically free of visible fibroblast growth within 3 passages (Fig. 1a, b). Cancer-associated fibroblasts from tumor specimens were readily isolated by plating sediments from digested tumor tissues on treated tissue culture plates. Fibroblasts selectively detached from tissue culture plates upon trypsinization for 2 min. These fibroblast cultures derived from tumor tissues expressed Vimentin and harbored no KRAS mutation as did the patient’s tumor (Fig. 1b), distinguishing them from epithelial tumor cells. Fibroblasts in initial organoid cultures were closely associated with organoids (Fig. 1d). Fibroblasts that attached to the well surface were able to be isolated from organoids growing in Matrigel domes for further propagation of tumor associated fibroblasts in 2D culture with primary cell culture media (Fig. 1a). The time to establishment of fibroblast cultures was typically 3 weeks from collection. Initial cultures of some organoids for extended periods of 2-3 weeks resulted in the outgrowth of monolayers of tumor cells on the surface of the wells. We were able to isolate and propagate these cultures as well, resulting in the establishment of 2D primary tumor cell cultures with relatively rapid separation from associated fibroblasts compared to direct 2D culture or PDX-derived methods.

**Table 1:** PDAC organoid culture overview

Organoid Cultures Initiated		Organoid Cultures Expanded and Banked		2D Cell Lines Derived		Matched CAF Cultures
37		28		3		2
Primary	Omentum/Peritoneum	Lung Met.	Liver Met.	Ascites	Autopsy	Normal Duct
21	2	1	2	1	2(1 + Met)	1

Peripheral Blood is also banked for all patients. 12 cultures have been fully characterized (markers and pathology)

**Table 2:** Characterization of organoids and cell lines

Tissue Bank #	Source	2D Culture	Days to Split	KRAS mutation	PDX1	CK19	Maspin	CRR9	αSMA	Vimentin
2112	primary	N	7			+		+		
1914	liver met	N	7			+		+		
2220	primary	N	3			+		+/−		
2423	lung met	Y	5	G12 V	+	+(+)	+(2.7)	+(3)	*-(−)*	–
1710	ascites	Y	1	G12D	–	+	+	+	–	–
RA012	rapid autopsy	Y	27	WT	+	+	-(−)	+	–	–
RA012 met	rap. Aut. met	N	20	WT	+	+		+	–	–
2534	omentum	N	3	G12D	+	+(+)	+(2.7)	+(3)	-(−)	–
2475	liver met	N	17	WT	–	+(+)	+(1.0)	+(3)	-(−)	–
2535	peritoneum	N	5	G12 V	+	+(+)	(2.7)	+(3)	-(−)	–
2423CAF	CAFs	Y		WT		–		–	+*(+)	+
RA012CAF	CAFs	Y		WT		–		–	+*	+

normal adj. = tumor adjacent normal tissue. Rap. aut. Met = metastatic tumor from rapid autopsy. Blank – not determined. *αSMA was positive is a subpopulation in co-culture with organoids. Tumor tissue staining indices are in parentheses (IHC: 1 = weak, 2 = moderate, 3 = strong, multiplied by percentage of positive cells)

A summary of the organoids and cell lines that were established, propagated and banked is given in Table 1.

### Characterization of organoid cultures

**Fig. 2:**
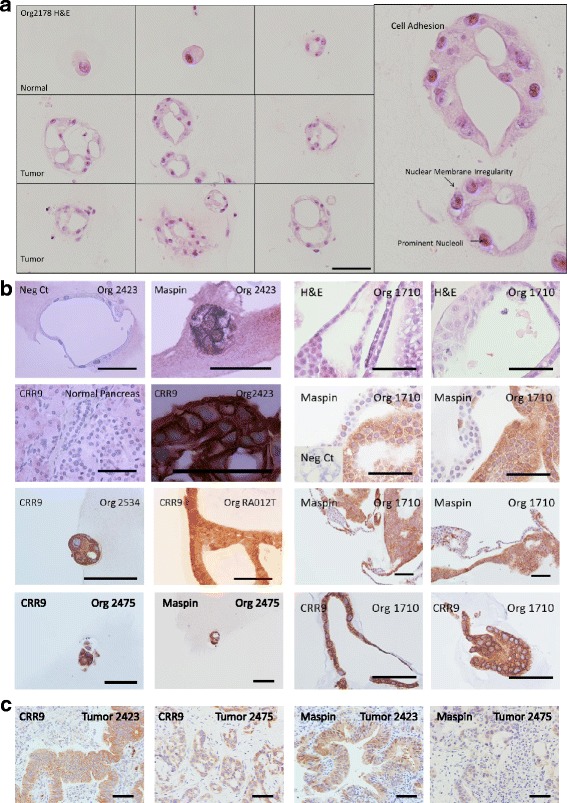
Pathologic and Immunohistochemical Characterization of Organoids. **a** H & E Staining and pathologic observations of organoid (tissue bank #2178). **b** Immunohistochemical staining with antibodies detecting the indicated proteins. Bars, 100 μM. **c** Immunohistochemical staining of tumor tissues

Propagation of tumor organoids in Matrigel by serial passage resulted in the selection of epithelioid populations demonstrating the tissue architecture and cellular morphology of ductal adenocarcinoma on H&E staining, namely, ductal structure with apicobasal polarity, cell adhesion, irregular nuclear membrane staining and prominent nucleoli confirming malignancy as determined by pathologist review (Fig. 2a). Normal ductal epithelial organoid cultures did not demonstrate nuclear irregularity, nucleolar prominence or cell-cell adhesion properties as tumor-derived organoids did.

**Fig. 3:**
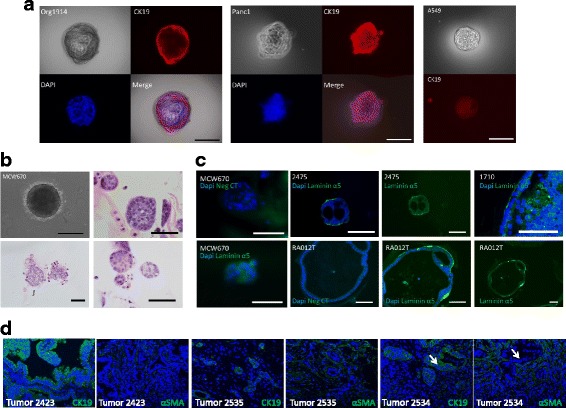
Immunofluorescent Characterization of Organoids. **a** IF staining of representative 1914 organoid and Panc1 spheroid as described using the indicated antibodies. **b** H&E on MCW670 spheroids. **c** IF staining of Laminin α5 in sections of 3D spheroids and organoids. Bars, 100 μM. **d** IF staining of CK19 in tumor tissues and αSMA in stromal fibroblasts. Arrows indicate CK19 positive tumor tissue surrounded by αSMA positive fibroblasts

Immunohistochemical and immunofluorescent evaluation was conducted to characterize organoid expression of tumor markers Maspin, Muc5ac and CRR9, pancreatic marker PDX1 and gastroenteropancreatic and hepatobiliary epithelial marker CK19 (Figs. 2b and 3). Maspin is a serpin-family serine protease inhibitor [18] that plays a critical role in embryogenesis [19] and is expressed in pancreatic ductal adenocarcinoma (96%) but not normal pancreatic tissues [20]. CRR9 (Cisplatin Resistance-Related protein 9) is a novel up-regulated tumor antigen in PDAC and other cancers that is not expressed on normal ductal epithelium and is pro-tumorigenic and anti-apoptotic, as we and others have demonstrated [21–26]. Immunohistochemical evaluation of these markers in tumor tissues from which organoids were derived revealed similar staining patterns and intensities observed in organoids (Fig. 2c, Table 2).

Spheroid cultures of an established cell line, Panc1, one of our patient-derived cell lines passaged as a murine xenograft, MCW670, and a lung tumor cell line, A549, formed spheroids without lumens in both complex organoid growth medium and RPMI1640, which were distinct from the structured organoids derived directly from patients (primary organoids) (Fig. 3a). All pancreatic cancer organoids that were evaluated stained positively for CK19 expression by immunofluorescence, while spheroids of lung tumor cells did not (Fig. 3a). Cell line MCW670 formed solid spheroids in 3D cultures grown in OGM with no luminal structure (Fig. 3b). Primary organoids demonstrated apicobasal polarity with Laminin α5 expression at the basal surface while MCW670 cell line spheroids did not (Fig. 3c). Aggressively growing organoids from ascites (1710) demonstrated Laminin α5 expression on the basal surface, but disorganized growth of tumor tissue into the lumen with lack of polarity in those cells. Primary organoids also differed from cell line organoids in that the Wnt3 pathway factors, other growth factors and TGFβ inhibitors in OGM were required for their anchorage independent growth, while cell line spheroids were able to grow in either OGM or RPMI1640 (data not shown).

Evaluation of tumor tissues from which organoids were derived demonstrated CK19 staining in all tumor tissues tested and αSMA (activated fibroblast marker) stained fibroblasts in the surrounding desmoplatic stroma. Again, immunofluorescence detection of markers in tumor tissues from which organoids were derived revealed similar staining patterns and intensities observed in organoids (Fig. 3d, Table 2).

KRAS exon 2 and 3 mutational hot spots were sequenced to determine codon 12 and 61 mutations using genomic DNA from early passage organoids cultures revealing codon 12 mutations in 5 of 7 patients tested. A summary of characterization of select organoid and cell line cultures is represented in Table 2.

### Development of 3D organotypic microenvironment models

**Fig. 4:**
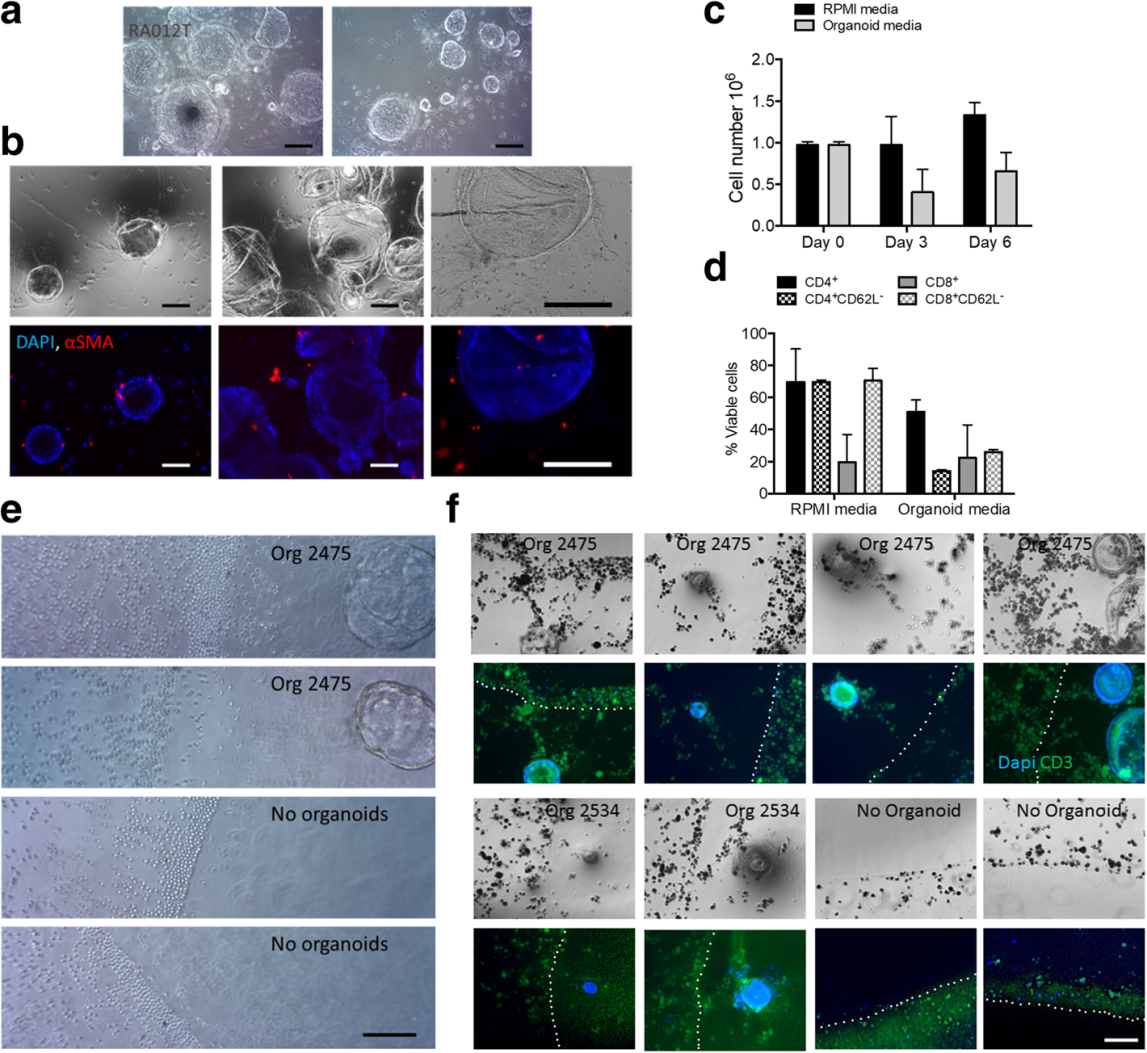
Co-culture of PDAC Tumor Organoids, CAFs, and Lymphocytes. **a** RA012 primary tumor organoids and CAFs. **b** Micrographs and immunofluorescence for the indicated markers on co-cultured RA012 primary tumor organoids and fibroblasts. **c** Number of viable cells in culture at different time points. **d** Percentage of T cell subsets in culture with CD3/26 activation on day 6. **e** Micrographs of T-lymphocytes in co-culture with organoids or empty Matrigel domes at the Matrigel boundary. **f** Micrographs of fixed cells and immunofluorescence using anti-CD3 and DAPI staining of T-lymphocytes in co-culture with organoids or empty Matrigel domes at the Matrigel boundary. Dotted lines represent Matrigel boundaries. Bars, 100 μM

Initial cultures of primary tumors included a mixed population of epithelioid organoids and closely associated cells with a morphology consistent with fibroblasts (Fig. 4a). Fibroblasts from tumor specimens of 3 patients were established and characterized as described in *Methods*. Wild-type KRAS (differed from tumor genotype for 2423 only) and Vimentin expression in fibroblasts (Table 2, Fig. 1b) confirmed no contamination by tumor epithelium. The expression of smooth muscle actin (αSMA) in a subset of fibroblasts, which is characteristic of myofibroblast-like activated pancreatic stellate cells, was detected by immunofluorescence in patient-matched CAFs in co-culture with human tumor organoids from a primary tumor obtained from a rapid autopsy (RA012) (Fig. 4b). Expression of αSMA was not detected by immunofluorescence in CAFs grown on plastic in monoculture (data not shown).

Human T-Cells isolated from the buffy coat by negative selection as described in *Methods* (Fig. 4c) were cultured under organoid culture growth conditions. CD4+ and CD8+ T-cells were viable and Ki67 positive upon treatment with CD3/CD28 beads in organoid growth media (OGM), although there was reduced viability in OGM compared to that in RPMI (Fig. 4c and data not shown). T-cells grown in OGM without organoids also demonstrated a less activated phenotype (CD62/L-selectin negative population) than those grown in RPMI (Fig. 4d). However, viable T-cells at representative CD4:CD8 ratios remained after 6 days in culture in OGM.

T-lymphocytes grown in the liquid phase of organoid cultures for 72 h were viable, and those juxtaposed to the boundary of empty Matrigel domes formed a distinct line, not infiltrating the Matrigel (Fig. 4e). However, T cells at the boundary of Matrigel domes containing patient-derived primary organoids infiltrated the Matrigel, migrating toward the organoids and diffusing the boundary. Immunofluorescent staining using anti-CD3 and DAPI demonstrated the same lymphocyte infiltration only in the presence of organoids (Fig. 4f). DAPI stained large tumor nuclei brightly, relative to non-activated, quiescent T-cells with condensed nuclei. These results indicate tumor dependent migration of T-cells in these novel organotypic models.

**Fig. 5:**
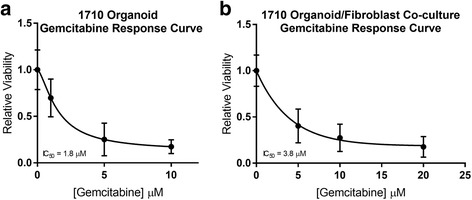
Gemcitabine Response in Organoid Co-Culture Versus Organoids Alone. **a** Relative viability in organoid cultures treated with indicated doses of gemcitabine for 96 h. **b** Relative viability in organoid-fibroblast co-cultures treated with indicated doses of gemcitabine for 96 h. Error bars indicate standard deviation from the mean

Co-culture of organoids with stromal cellular components appeared to have an effect on resistance to 96 h gemcitabine treatment, as growth of PDAC organoids in co-culture with CAFs resulted in an increased IC50 of 3.8 μM compared to 1.8 μM with organoids alone (Fig. 5).

## Discussion

There is a stark absence of studies in the literature using in-vitro models of the pancreatic adenocarcinoma microenvironment. This is, perhaps, surprising, given the known importance of both stromal and immune interactions with pancreatic tumors. While organoid or spheroid culture is becoming an attractive and increasingly utilized technique, it is important to note that 3D anchorage independent culture of established cell lines propagated in monolayers or those derived from mouse xenografts are distinct in character from primary organoids derived directly from patient specimens. Primary organoids retain the tumor tissue architecture, cellular heterogeneity in terms of mutational profile and stemness, cell-cell interactions, and polarity of tumor tissue in-situ. Cell lines that have emerged from the selective pressures of xenograft and/or adherent culture display a lack of structure and polarity, and much more quickly and readily grow and passage perpetually as spheroids than primary tumor organoids. We emphasize that organoids from xenografts and established cell lines do not closely represent in-situ tumors as distinct from primary patient-derived organoid cultures, which do.

**Fig. 6:**
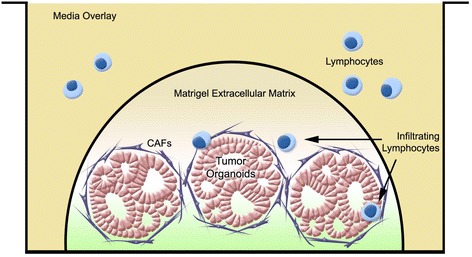
Schematic representation of organotypic organoid co-cultures

We have developed complex, patient-matched, organotypic models incorporating human pancreatic cancer organoids, CAFs and T-cells (Fig. 6). Models such as these have the potential to become a paradigm for precision modeling and guidance of personalized medicine. Our complex 3D organotypic models may also be amenable to bioprinting technologies to precisely structure the tumor architecture in these cultures.

In agreement with recent findings from mouse pancreatic cancer organoid and fibroblast co-culture [8], we observed expression of αSMA, indicating an activated myofibroblast-like phenotype, in only a subset of fibroblasts grown with tumor organoids. Interestingly, CAFs grown on plastic in monoculture did not express αSMA, contrary to the observations of Jesnowski et al. [27]. The work with mouse organoids also strengthened available evidence that fibroblasts contribute to tumor survival and growth, probably through paracrine cytokines such as IL-6. These in-vitro organotypic models incorporate this important paracrine signaling. In the present study, we expand upon these findings by inclusion of immune cells to organoid and CAF co-cultures. We have demonstrated that these multi-cell type organotypic co-cultures are a viable technique for the in-vitro study of pancreatic cancer-associated stromal and immune interactions as well as models of the tumor microenvironment.

One of the more attractive applications for these models is precision testing of drug response. These models can be rapidly established using a patient’s own tissues. Although we do not describe the culture of organoids from fine-needle aspiration (FNA) biopsy specimens in this report, such techniques have been accomplished [15], and we are pursuing the utilization of organoids from FNA biopsies as a means to rapidly evaluate genetic and phenotypic markers as well as to facilitate target identification and potentially guide the delivery of individualized therapy. For example, we have just completed enrollment of a clinical trial using molecular profiling to guide the choice of chemotherapy in patients with resectable and borderline resectable pancreatic cancer. While tissues and models that are representative of the patient’s disease hold promise for the accurate prediction of drug response, the complexity of tumor tissue acquisition and the long-term and unreliable nature of establishing patient-derived xenograft avatars make these resources unattractive for widespread application. In contrast, implementing organoid models in clinical trials of personalized medicine may represent the technology of choice.

The incorporation of lymphocytes into pancreatic cancer organotypic cultures is particularly relevant to the burgeoning field of immunotherapeutics, especially immune checkpoint inhibition. We have demonstrated the ability to observe infiltration of the extracellular matrix surrounding tumor tissues in these models. This represents a novel model of tumor-immune interaction in pancreatic cancer that may possibly be extrapolated to other cancer types. Much of the Chemokines such as CXCL12 expressed by tumor organoids may induce this migration, although it is possible that effects of tumor organoids on degradation of the extracellular protein matrix may contribute to this observation. Further mechanistic study of immune infiltration into human pancreatic cancer using models such as we describe is warranted. Much of the seminal nature of this study is in the establishment of models for immuno-oncology. We anticipate that these models will be a highly valuable method of rapidly evaluating the effect of checkpoint inhibition on the activation and infiltration of cytotoxic lymphocytes in tumor tissues in the context of T-cell infiltrates. Further work is required to investigate the incorporation of other cells involved in tumor immunity, such as dendritic and myeloid-derived suppressor cells.

## Conclusion

In conclusion, we demonstrate novel and disease-relevant 3D in-vitro models representing human pancreatic cancer to include stromal and immune components using primary organoid co-cultures that are amenable to the study of tumor-microenvironment and tumor-immune cell interaction.

## Abbreviations

CAF: Cancer-associated fibroblast
CK19: Cytokeratin 19
CLPTM1L/CRR9: Cleft lip and palate transmembrane 1-like/Cisplatin resistance related protein-9
ECM: Extracellular matrix
OGM: Organoid growth media
PDAC: Pancreatic ductal adenocarcinoma
PDX: Patient-derived xenograft
PDX-1: Pancreatic and duodenal homeobox 1
TIL: Tumor-infiltrating lymphocyte
αSMA: alpha-smooth muscle actin
